# Synthetic Microbial Community Isolated from Intercropping System Enhances P Uptake in Rice

**DOI:** 10.3390/ijms252312819

**Published:** 2024-11-28

**Authors:** Huimin Ma, Hongcheng Zhang, Congcong Zheng, Zonghui Liu, Jing Wang, Ping Tian, Zhihai Wu, Hualiang Zhang

**Affiliations:** 1 Faculty of Agronomy, Jilin Agricultural University, Changchun 130118, China2201050203@mails.jlau.edu.cn (Z.L.);; 2 ISPA, INRAE, F-33140 Villenave d’Ornon, France; zhengcc342@gmail.com; 3 State Key Laboratory of Vegetation and Environmental Change, Institute of Botany, Chinese Academy of Sciences, Beijing 100093, China; jing_wang43@ibcas.ac.cn; 4 China National Botanical Garden, Beijing 100093, China; 5 Institute of Soil and Water Resources and Environmental Science, College of Environmental and Resource Sciences, Zhejiang Provincial Key Laboratory of Agricultural Resources and Environment, Zhejiang University, Hangzhou 310058, China

**Keywords:** intercropping, rice, synthetic microbial community, root morphology, P uptake

## Abstract

Changes in root traits and rhizosphere microbiome are important ways to optimize plant phosphorus (P) efficiency and promote multifunctionality in intercropping. However, whether and how synthetic microbial communities isolated from polyculture systems can facilitate plant growth and P uptake are still largely unknown. A field experiment was first carried out to assess the rice yield and P uptake in the rice/soybean intercropping systems, and a synthetic microbial community (SynCom) isolated from intercropped rice was then constructed to elucidate the potential mechanisms of growth-promoting effects on rice growth and P uptake in a series of pot experiments. Our results showed that the yield and P uptake of intercropped rice were lower than those of rice grown in monoculture. However, bacterial networks in the rice rhizosphere were more stable in polyculture, exhibiting more hub nodes and greater modularity compared to the rice monoculture. A bacterial synthetic community (SynCom) composed of four bacterial strains (*Variovorax paradoxus*, *Novosphingobium subterraneum*, *Hydrogenophaga pseudoflava*, *Acidovorax* sp.) significantly enhanced the biomass and P uptake of potted rice plants. These growth-promoting effects are underpinned by multiple pathways, including the direct activation of soil available P, increased root surface area and root tip number, reduced root diameter, and promotion of root-to-shoot P translocation through up-regulation of Pi transporter genes (*OsPht1;1*, *OsPht1;2*, *OsPht1;4*, *OsPht1;6*). This study highlights the potential of harnessing synthetic microbial communities to enhance nutrient acquisition and improve crop production.

## 1. Introduction

Rice (*Oryza sativa* L.) is a staple food consumed by more than half of the world’s population and is the third most important cereal globally after maize (*Zea mays* L.) and wheat (*Triticum aestivum* L.) [1,2]. It is also a water-intensive crop and is highly sensitive to drought [3]. Traditional waterlogged rice cultivation requires significant amounts of freshwater [4,5]. With the rapid global population growth and increasing water scarcity, drought has become a significant threat to rice yields. A novel cultivation system for rice in aerobic soil is considered a promising water-saving model in agriculture [6,7]. Additionally, cultivating rice in aerobic soil allows for intercropping with legumes. As a promising agricultural practice for stabilizing the crop yield, rice/legume intercropping offers numerous advantages, including improved soil fertility and increased water use efficiency.

Cereal/legume intercropping has been a widespread practice in recent decades and is now attracting increasing interest as sustainable agricultural practices are developed [8]. Numerous previous studies have reported that intercropping leads to significantly greater yield and higher P acquisition of the stronger competitors in maize/alfalfa, wheat/faba bean, and durum wheat/chickpea intercropping systems [9,10,11]. The enhancement of plant P acquisition and yield production in intercropping can be attributed to two pathways: (i) a root-mediated pathway and (ii) a microbiota-mediated pathway [12,13]. The root-mediated pathway involves the ability of plant roots to enhance rhizosphere P availability by excreting P-mobilizing compounds, including protons, organic acids, carboxylates, and phosphatases [14,15]. It also includes optimized root system architecture for P uptake though increasing root surface area, fine root density and root tip numbers, as well as promoting shallower root growth angles [11,16]. By contrast, the microbiota-mediated pathway plays a key role in P cycling through the symbiotic microbiome near living roots [12]. In the rhizosphere of stronger competitors, enriched phosphorus-solubilizing microbes significantly dissolve mineralized P, thus increasing soil P availability [17,18]. While stronger competitors can acquire more P through both pathways, few studies have examined these pathways in weaker competitors, particularly regarding the role of microbiota-mediated effects on plant P uptake.

It is well known that root-associated bacteria in the intercrop rhizosphere can be classified as plant growth-promoting rhizobacteria (PGPR), which may perform a number of essential biological functions, such as improving plant nutrient acquisition, enhancing pathogen tolerance, and increasing stress tolerance [19]. Under P stress, PGPRs have been shown to improve P uptake in multiple plant species by optimizing root morphology, often induced by bacterial metabolites such as hormones (e.g., auxin) or signaling compounds [20,21]. In addition, the discovery of several PGPRs, including *Variovorax*, *Acidovorax*, and *Hydrogenophaga*, that dissolve P in soil by lowering soil pH through the microbial production of organic acids and acid phosphatase has highlighted their potential role in enhancing P availability [22]. Recently, synthetic microbial communities (SynComs) have attracted considerable attention under climate change. Compared with a sole microbiome, a SynCom allows the simplification and simulation of natural microbial communities, which offers an important platform to explore the functional mechanisms of plant microbiota, such as PGPRs [23,24]. Meanwhile, traditional biotechnological approaches predominantly rely on single microbial inoculants, which, while easy to formulate, often exhibit limited competitiveness and are constrained by factors such as stability, dosage, efficacy, target specificity, and sensitivity to application environments. In contrast, a SynCom leverages synergistic interactions among microbial members, resulting in enhanced competitiveness and functionality [25,26]. This makes SynComs a promising alternative to single-strain inoculants, with the potential to address numerous challenges faced by modern agriculture. However, our understanding of how these phosphate-solubilizing bacteria regulate root distribution and morphology remains limited.

In this article, we hypothesize that assembling and applying a SynCom comprising enriched strains in intercropped rice roots under intense interspecific competition can enhance rice P uptake by improving soil P availability, optimizing root morphology, and facilitating root-to-shoot P translocation. Our aims are (1) to identify how intercropping with soybean affects rice biomass and P uptake, (2) to evaluate the effectiveness of the SynCom in promoting P uptake in rice, and (3) to elucidate the potential mechanisms of the SynCom in conferring improved plant P uptake.

## 2. Results

### 2.1. Intercropping Modulates Rice Growth, P Uptake and Soil Microbiota in Field Experiment

**Figure 1:**
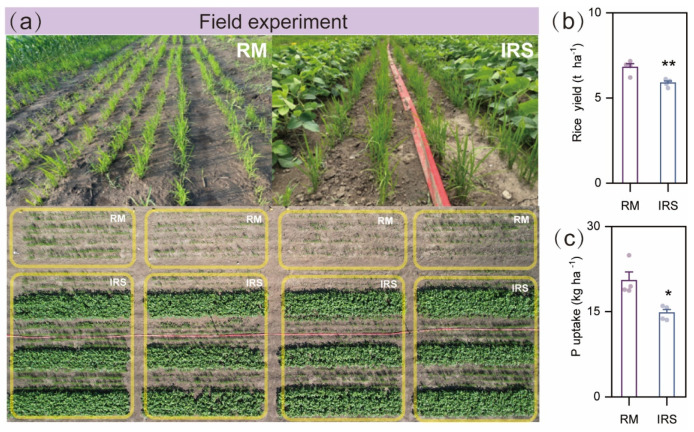
Intercropping inhibits rice growth under field conditions. (**a**) Diagram of the field experiment design and growth phenotype of rice plants in rice monoculture and intercropping treatment. RM: rice monoculture, IRS: rice/soybean intercropping treatment. (**b**,**c**) Rice yield and P uptake of rice plants grown in monoculture and intercropping under field conditions (average ± s.e.m, Rice yield and P uptake: *n* = 4. Individual replicate samples are represented by data points. Significant differences between treatments are indicated by different letters or asterisks (ANOVA, FDR-corrected LSMeans, *, *p* < 0.05; **, *p* < 0.01).

Intercropping significantly modified and inhibited rice growth, P uptake and soil microbiota of rice in the intercropped rice/soybean (IRS) treatment in northern China. Our results showed that intercropping with soybean significantly decreased the yield and P uptake of rice compared to the rice monoculture by 13.6% and 28%, respectively (Figure 1b,c).

**Figure 2:**
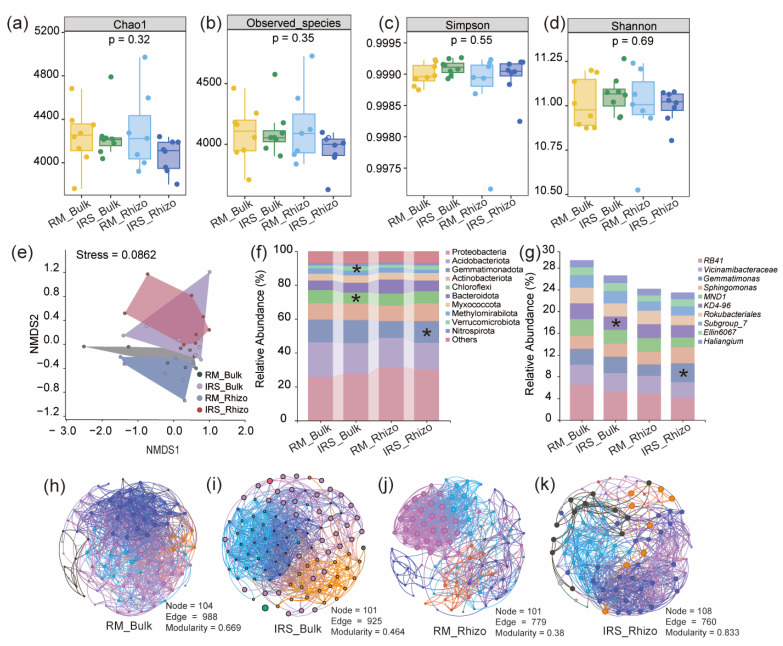
Intercropping changes microbial community composition. (**a**–**d**) Bacterial alpha diversity of Chao1, Observed species, Simpson and Shannon index. (**e**) Non-metric multi-dimensional scaling (NMDS) analysis of bacterial communities in bulk soil (RM_Bulk and IRS_Bulk) and rhizosphere soil (RM_Rhizo and IRS_ Rhizo) of rice plants grown in monoculture and intercropped soil (bulk: *n* = 4, rhizosphere: *n* = 4). (**f**,**g**) Phylum and genus level distribution of bacteria communities in the bulk and rhizosphere of rice plants grown in rice monoculture and intercropping soils (bulk: *n* = 4, rhizosphere: *n* = 4). Asterisks indicate significant differences between treatments (ANOVA, FDR-corrected LSMeans, *, *p* < 0.05). (**h**–**k**) Co-occurrence patterns of microbial communities in bulk soil (RM_Bulk and IRS_Bulk) and rhizosphere soil (RM_Rhizo and IRS_ Rhizo) of rice plants grown in monoculture and intercropping soil (bulk: *n* = 8, rhizosphere: *n* = 7–8).

To examine the impact of intercropping on root-associated microbiota community composition, we profiled the bacterial communities in the bulk soil and rhizosphere of rice plants grown in both monoculture and intercropping systems. Compared with monoculture, rice/soybean intercropping had no effect on the Chao1, Observed species, Simpson and Shannon indicators of bacterial α-diversity in the rice rhizosphere (Figure 2a–d). However, non-metric multi-dimensional scaling (NMDS) analysis based on Bray–Curtis distances showed that intercropping significantly altered the overall composition of root and rhizosphere bacterial communities (Figure 2e). At the phylum level, intercropping increased the relative abundance of Verrucomicrobiota in the bulk soil and Myxococcota in the rhizosphere of rice plants (Figure 2f). At the genus level, only *Gemmatimonas* was observed to be enriched in the rhizosphere of rice plants (Figure 2g)

To assess how intercropping influences microbial networks, we examined the co-occurrence networks of microbial communities using relative ASV abundances. Significant differences in the topological characteristics of bacterial networks were noted between monoculture rice and intercropped rice (Figure 2h–k; Table S1). Notably, intercropped rice exhibited greater modularity and a higher number of nodes in the rhizosphere soil compared to rice grown in monoculture. These results demonstrate that intercropping markedly alters soil microbiota composition.

**Figure 3:**
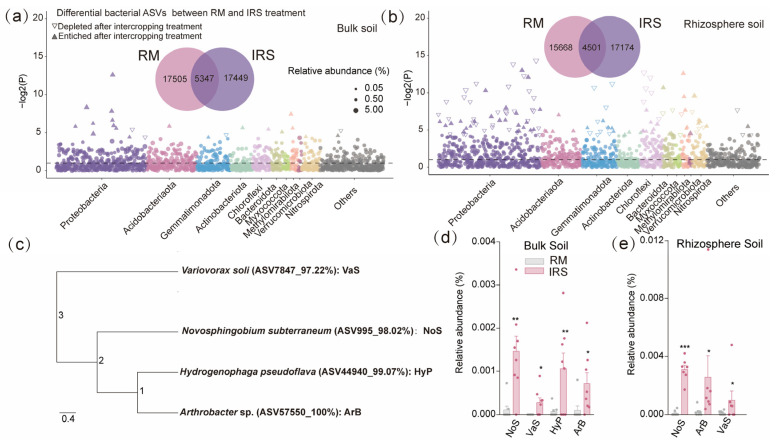
Intercropping enriched specific rhizosphere-associated bacteria in rice plants. (**a**,**b**) Manhattan plot of ASV-enriched bacteria in the bulk and rhizosphere soil of rice plants growing in in rice monoculture and rice/soybean intercropping mode soils. (**c**) Phylogenetic trees based on cultured bacterial strains isolated from rice grown in monocropping and rice/soybean intercropping soils. (**d**,**e**) Similarity (>97%) of the four selected strains with detected ASVs, showcasing their relative abundances in bulk and rhizosphere soil. In (**a**,**b**) each dot or triangle represents a single ASV. ASVs enriched or depleted in intercropping soil are represented by filled or empty triangles, respectively (FDR adjusted *p* < 0.05, DESeq2). Significant differences between treatments are indicated by different asterisks (ANOVA, FDR-corrected LSMeans, *, *p* < 0.05; **, *p* < 0.01; ***, *p* < 0.001).

To elucidate the growth-enhancing effects of specific enriched microbiota in the root-associated soil of rice, we compared the differences in bacterial ASVs (relative abundance > 0.1%) between monoculture and intercropping treatments. Intercropping significantly enriched 21 ASVs in bulk soil and 76 ASVs in rhizosphere soil, while depleting 4 ASVs in bulk soil and 78 ASVs in rhizosphere soil (Figure 3a,b). Subsequently, bacteria were isolated from both bulk and rhizosphere soils under monoculture and intercropping conditions. Those with >97% 16S rRNA gene similarity to detected ASVs were selected for further experiments. Four isolates were selected from enriched genera that closely matched their corresponding ASV sequences; i.e., *Novosphingobium subterraneum* (ASV995, 98.02%), *Hydrogenophaga pseudoflava* (ASV44940, 99.07%), *Acidovorax* sp. (ASV57550, 100%), and *Variovorax paradoxus* (ASV7847, 97.22%).

### 2.2. Synthetic Community Improves Growth and P Uptake of Rice in Pot Expriment

**Figure 4:**
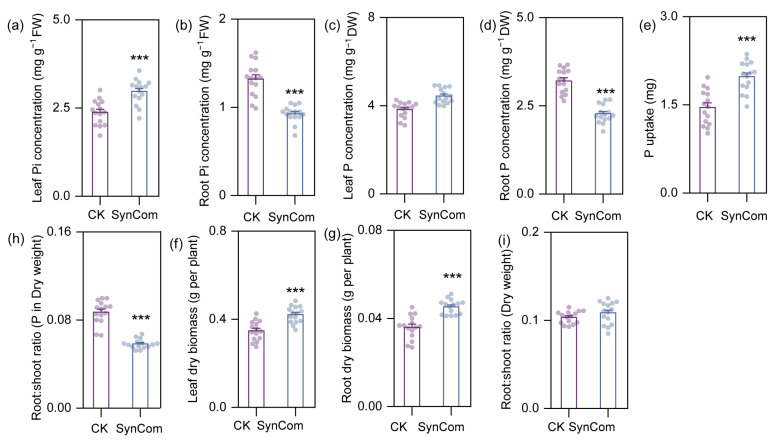
Inoculation with SynCom affects biomass and P uptake of rice plants. (**a**–**i**) leaf Pi concentration, root Pi concentration, leaf P concentration, root P concentration, P uptake, root:shoot ratio of P uptake, leaf dry biomass, root dry biomass and root:shoot ratio of dry biomass of rice treated with SynCom and sterilized water (average ± s.e.m, *n* = 15). Significant differences between treatments are indicated by different asterisks (ANOVA, FDR-corrected LSMeans, ***, *p* < 0.001).

SynCom inoculation significantly increased Pi concentration of rice shoots in pot experiments, while reducing Pi concentration and P concentration in roots, suggesting that the SynCom regulated aboveground and belowground P allocation in rice (Figure 4a,b). Additionally, the reduced root:shoot ratio of P uptake with SynCom inoculation further confirmed increased P transfer from root to shoot compared to the control. Concomitantly, SynCom inoculation significantly increased both leaf and root dry biomass without affecting the root-to-shoot biomass ratio (Figure 4f–i).

### 2.3. SynCom Participates in the Activation of Soil Phosphorus and the Modification of Root Morphology

**Figure 5:**
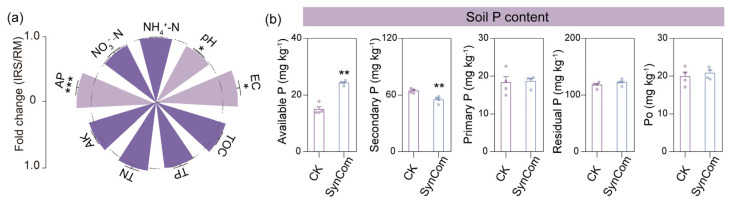
Inoculation with SynCom affects rhizosphere soil quality of rice. (**a**) Soil properties are depicted as fold changes in SynCom-treated soil relative to sterilized water-treated soil (average ± s.e.m, *n* = 4). For the full datasets, refer to Figure S1. (**b**) Different forms and fractions of P in the soil of planted rice inoculated with sterilized water or SynCom (average ± s.e.m, *n* = 4). Significant differences between treatments are indicated by different asterisks (ANOVA, FDR-corrected LSMeans, *, *p* < 0.05; **, *p* < 0.01; ***, *p* < 0.001).

SynCom significantly increased electrical conductivity (EC) and available phosphorus levels in the rice rhizosphere while lowering pH (Figure 5a). Further analysis of soil P fractions showed that SynCom inoculation significantly increased available P content in the soil by 62.5%, while reducing secondary P by 14.6%, with no significant effects on other P forms (Figure 5b). Together, the recruitment of specific bacteria can directly promote P uptake by facilitating the transformation of secondary P into available P in the soil.

**Figure 6:**
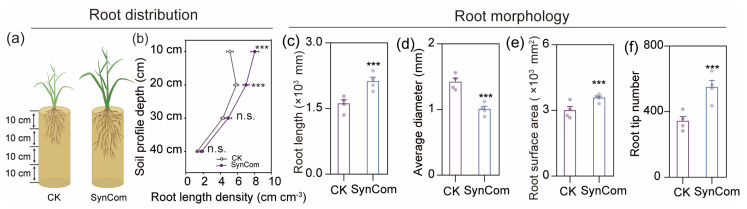
Inoculation with SynCom affects root distribution and root morphology of rice plants. (**a**) Schematic diagrams of potted rice. (**b**) Root length density of rice treated with SynCom and sterilized water. *n* = 4 biological replicates were measured for all treatments. Data are average ± s.e.m. (**c**–**f**) Root length, average diameter, root surface area, root tip number of rice plants inoculated with sterilized water (CK) or bacterial synthetic community (SynCom). *n* = 4 biological replicates were measured for all treatments. Data are average ± s.e.m. Data points represent individual replicate samples. Asterisks indicate significant differences between treatments (ANOVA, FDR-corrected LSMeans, ***, *p* < 0.001; n.s., not significant).

Furthermore, SynCom inoculation was observed to exert a significant impact on the root distribution and morphology of rice, when compared to the control. Consistent with our hypothesis, SynCom inoculation significantly promoted root length density in the topsoil (0–20 cm) by 36.5% and also increased root length, surface area and tips while reducing root diameter (Figure 6b–f).

### 2.4. SynCom Alters Phosphate Transport from Root to Leaf in Rice Plant

**Figure 7:**
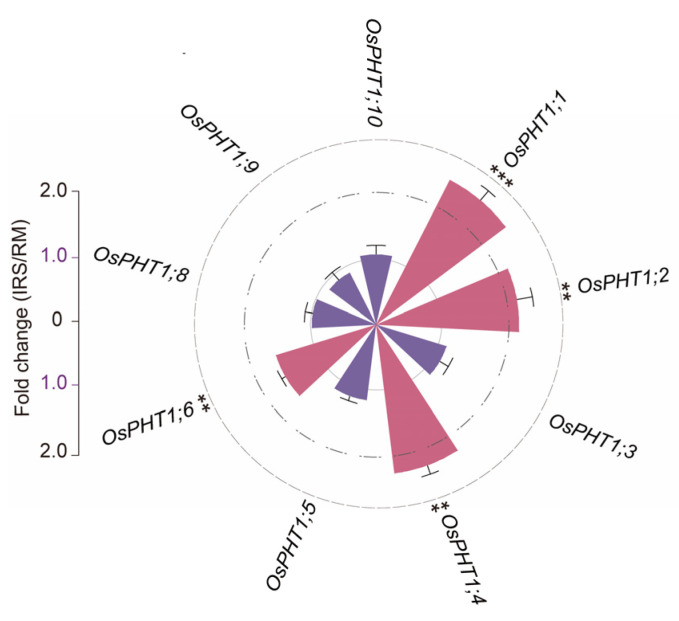
Inoculation with SynCom affects P transporters. SynCom affects the expression of PHT1 family transporter genes related to root-to-shoot Pi translocation in rice leaves and roots. For the full datasets, refer to Figure S3 Significant differences between treatments are indicated by different asterisks (ANOVA, FDR-corrected LSMeans, **, *p* < 0.01; ***, *p* < 0.001).

To further understand the molecular mechanisms underlying the alteration of P transport by the SynCom, we profiled the expression of PHT1 family transporter genes related to root-to-shoot Pi translocation in rice roots inoculated with SynCom. A significant increase in the expression of *OsPht1;1*, *OsPht1;2*, *OsPht1;4*, and *OsPht1;6* was observed; these genes are involved in root-to-shoot Pi translocation and more Pi accumulation in the shoot (Figure 7). Thus, the SynCom enhances P uptake of leaf via promoting P transport from root to shoot.

## 3. Discussion

### 3.1. Interspecies Competition Inhibits Yield and P Uptake of Rice Plants in the Rice/Soybean Intercropping System

The enhancement of P uptake from P-limited soils and overall yields through increased crop diversity is an increasingly recognized strategy with significant potential [27]. A number of greenhouse and field experiments have proved that the intercropping advantage mainly depends on the overyielding and high P uptake of the dominant species in maize/alfalfa, wheat/faba bean, and durum wheat/chickpea intercropping systems [9,10,11]. It is also reported that the intercropping advantages of rice/soybean are primarily attributed to overyielding of soybean, with a slight sacrifice in rice yield [28]. Consistent with previous studies, our results reveal that intercropping significantly inhibited rice yield and P uptake in field experiments (Figure 1b,c). Although the total yield and nutrient uptake of intercropping can be higher than those of sole systems, our results showed that rice exhibited a competitive disadvantage when grown with soybean.

### 3.2. Rice/Soybean Intercropping Reshapes Rhizosphere Bacterial Communities of Rice Plants

It has been well documented that multiple crops can actively reshape rhizosphere bacterial communities to respond to different environmental stresses when intercropping with other crops, such as promoting nutrient acquisition, antagonizing pathogens, and enhancing drought tolerance [29,30,31]. Here, our results showed that intercropping had no effect on microbial alpha diversity in the bulk and rhizosphere soil of rice, while it significantly shifted microbial community composition (Figure 2a–g). Furthermore, we found that intercropping had a significant influence on the rhizosphere microbial co-occurrence network of rice, with a greater number of nodes and higher modularity. These results are consistent with previous research demonstrating that intercropping increases the nodes, edges, and modularity of bacterial networks in the cereal rhizosphere [32,33]. A more stable microbial community can be induced by special root exudates released by neighboring legume crops, such as lectins, flavonoids, naringenin, and isoflavones, which further contribute to overyielding in intercropping systems [34,35,36].

### 3.3. SynCom Promotes Yield and P Uptake of Rice Plants by Multiple Pathways

By analyzing the differences in root-associated microbes between monoculture and intercropping, we found that intercropping significantly enriched 21 ASVs in bulk soil and 76 ASVs in rhizosphere soil, while depleting 4 ASVs in bulk soil and 78 ASVs in rhizosphere soil (Figure 4a,b). Subsequently, we further selected four isolates of enriched bacterial strains to build a synthetic community (SynCom), namely *Variovorax paradoxus*, *Novosphingobium subterraneum*, *Hydrogenophaga pseudoflava*, and *Acidovorax* sp. The SynCom significantly promoted the growth and P uptake of the rice root (Figure 4); this is consistent with previous findings that inoculation with a SynCom composed of Al-tolerant PGPRs can be used as a key means to increase rice Al toxicity resistance and P uptake [30].

We further explored the mechanisms behind the growth-promoting effect of the SynCom on rice during the seedling stage. First, we evaluated the effectiveness of the SynCom in altering soil nutrient content. SynCom inoculation significantly reduced soil pH in alkali soil while increasing available P concentration in the rice rhizosphere (Figure 5a). Similar results have also been reported in other intercropping systems [9,37]. The potential mechanism likely involves a decrease in pH due to the release of lactic acid, acetic acid, and citric acid from rhizosphere bacteria (e.g., *Variovorax paradoxus*), which solubilize soil-insoluble phosphates and enhance their bioavailability for plant growth [38,39,40]. Further analysis of soil P fractions showed that SynCom inoculation significantly increased available P content in the soil by 62.5%, while reducing secondary P by 14.6% (Figure 5b). This suggests that the SynCom facilitates the dissolution of soil P minerals (such as Ca–P, Al–P, Fe–P, and O–P) [41], which is consistent with previous studies indicating that beneficial bacterial inoculation can convert soil insoluble organic P or inorganic P into plant-absorbable forms [42]. For example, *Variovorax paradoxus*, as a representatives of P-cycling bacteria, can produce alkaline phosphomonoesterase and dissolve inorganic P to increase its bioavailability [40]. *Novosphingobium barchaimii* exhibits tricalcium phosphate solubilizing activity [43]. *Arthrobacter* sp. is considered a major decomposer of organophosphorus, including phosphonates and phosphate esters [44]. Together, our result confirms that a SynCom comprising phosphate-solubilizing bacteria increases soil P bioavailability for plant absorption and utilization.

Additionally, SynCom inoculation modified root distribution in the shallow layers (0–20 cm) (Figure 6b). Due to the low solubility and mobility of P in soil, it tends to accumulate in the topsoil [39]. This enhanced root distribution in topsoil can increase P uptake by rice. More importantly, SynCom inoculation optimized root morphology with greater root length, surface area, and number of root tips, while producing thinner roots (Figure 6c–f). This can be attributed to secretion of auxins or auxin-like compounds by plant growth-promoting rhizobacteria, which are involved in the regulation of root development during plant–microbe interactions [45]. For instance, *Acidovorax* sp., *Hydrogenophaga* sp., and *Novosphingobium* sp., as main IAA producers, promote root elongation and stimulate lateral and adventitious root growth [46,47,48]. Consistent with these studies, we found that SynCom inoculation significantly increased indole-3-acetic acid (IAA) content in rice roots (Figure S2). Thus, SynCom inoculation improved P uptake through enhanced root distribution in the topsoil and modified root morphology in rice.

SynCom inoculation remarkably improved shoot Pi content while decreasing root Pi content compared to the control (CK), resulting in a higher shoot-to-root ratio of P uptake. This suggests that the SynCom enhanced long-distance Pi transport from roots to shoots, thereby boosting shoot P uptake. This mechanism likely involves the activation of Pi transport genes in rice roots, particularly PHT1 family transporters, which play a critical role in root-to-shoot P translocation [49]. These genes can facilitate the transport of Pi from roots to the stems, leaves and seeds of the aboveground parts. With a quantitative analysis of P transport-related genes, including PHT1 family transporters, in rice roots inoculated with SynCom, we observed a significant upregulation of *OsPht1;1*, *OsPht1;2*, *OsPht1;4*, and *OsPht1;6* in rice root. This is consistent with previous studies showing that higher expression levels of *OsPHT1;1*, *OsPHT1;4*, *OsPHT1;6* and *OsPHT1;8* are associated with greater Pi accumulation in shoots, with an increased root-to-shoot Pi ratio [50,51,52,53]. Thus, SynCom inoculation enhances P uptake and Pi translocation from roots to shoots in rice.

## 4. Materials and Methods

### 4.1. Soil and Plants

Soil samples were collected from an experimental agricultural field at Jilin Agricultural University, Jilin, China (125°40′ N, 43°81′ E). The paddy soil had a pH value of 7.2, containing 1.7 g kg^−1^ of total nitrogen (N), 17.2 g kg^−1^ of organic matter, 16.5 mg kg^−1^ available P, and 112 mg kg^−1^ available potassium (K). Soil samples were collected from the field and subsequently sieved through a 2 mm mesh to remove large stones and plant debris, and then stored in a shaded area in preparation for greenhouse trials conducted as described below. Rice (*Oryza sativa* L.) cultivar Suijing No. 18 was used in both greenhouse and field experiments, while soybean (*Glycine max* L.) cultivar Jinong No. 89 was selected as the intercropped legume species.

### 4.2. Field Experiment

The experiment was conducted at the Research Base of Jilin Agricultural University, Changchun, Jilin Province, Northeast China (125°40′ N, 43°81′ E, 238.6 m above sea level), with two cropping systems: rice monoculture (RM) under dry cultivation planted in eight rows (2.4 m × 6 m); (2) four rows of rice under dry cultivation intercropped with four rows of soybean, with a row spacing of 30 cm between maize and soybean strips (IRS) (7.2 m × 6 m). Rice under dry cultivation and soybean had seeding rates of 150 kg ha^−1^ and 200,000 plants ha^−1^, respectively, in early May 2023. Each treatment had four biological replicates. The field experiment was managed using local conventional tillage practices. At maturity (early October), the grain yield and above-ground and below-ground dry biomass of rice were measured by harvesting the first two central rows adjacent to the intercropped soybean. Four replicates, each containing five representative plants, were selected for both monoculture and intercropping treatments. All oven-dried samples were finely ground for P analysis. The P concentration was determined using the vanadomolybdate method following digestion of the plant material with a mixture of concentrated H_2_SO_4_ and HClO_4_.

### 4.3. Microbiota Profiling and Inoculation

To characterize root-associated microbiota in intercropping, we examined the bacterial community in both bulk and rhizosphere soils of rice plants from the rice monoculture or rice/soybean intercropping treatment in the field experiment. The four treatments included bulk soil (RM_Bulk) and rhizosphere soil (RM_Rhizo) from the rice monoculture, and bulk soil (IRS_Bulk) and rhizosphere soil (IRS_Rhizo) from the intercropping treatment. A total of eight bulk soil samples (four plots × two biological replicates) and eight rhizosphere soil samples (four plots × two biological replicates) were collected from the rice monoculture and intercropping treatments in August 2023. Three soil samples were combined to create one composite sample for each biological replicate. Rhizosphere soils were collected using a slightly modified method [54]. Briefly, sterile Silwet L-77 amended PBS buffer (PBS-S; 130 mM NaCl, 7 mM Na_2_HPO_4_, 3 mM NaH_2_PO_4_, pH 7.0, and 0.02% Silwet L-77) was used to wash the roots of gently uprooted rice plants. The rhizosphere soil was obtained by centrifuging the washing buffer containing detached soil particles. Bulk soil from each pot was also collected. These samples were divided into two portions: one for bacterial isolation and the other for microbiome analysis, which was stored at −80 °C.

The total soil genomic DNA was extracted from a 0.25 g soil sample following the standardized protocol suggested in FastDNA spinkits (MP Biomedicals, Solon, Ohio, USA). The V3–V4 regions of 16S rRNA genes were amplified using specific primers (338F: ACTCCTACGGGAGGCAGCA and 806R: GGACTACHVGGGTWTCTAAT) [55]. DNA library preparation and 16S rRNA gene sequencing were conducted by Personalbio Biotechnology Company (Shanghai, China). Taxonomic profiling of the soil microbiota was performed using DADA2 and QIIME2. Community α-diversity indexes (Chao1, Observed species, Simpson, and Shannon) based on 16S rRNA gene amplicon sequencing were calculated, and β-diversity was analyzed using non-metric multi-dimensional scaling (NMDS) with Bray–Curtis dissimilarity. The DESeq2 was used to analysis differences in bacterial ASV abundance between monoculture and intercropping systems, with *p*-values adjusted using False Discovery Rate (FDR), and then difference results were visualized using Manhattan plots. Co-occurrence network analysis evaluated species associations based on Spearman’s rank correlation, and only ASVs (relative abundance > 0.1%) were selected for network construction. The resulting network was visualized using Gephi (v0.1.0) with the following conditions: |r| > 0.6 and *p*-value < 0.05, and relevant topological parameters and node scores were determined. The raw sequencing data of soil microbiota are available in the Genome Sequence Archive in the National Genomics Data Center, China National Center for Bioinformation/Beijing Institute of Genomics, Chinese Academy of Sciences (GSA: CRA020594), publicly accessible at https://ngdc.cncb.ac.cn/gsa.

Plant-associated bacteria were isolated from the rhizosphere and bulk soil of rice grown under intercropping treatment [56]. The bacterial isolates were identified using 16S rRNA sequencing with primers 27F (5′-AGAGTTTGATCCTGGCTCAG-3′)/1492R (5′-GGTTACCTT GTTACGACTT-3′) following this protocol: initial denaturation at 98 °C for 3 min, followed by 30 cycles of 98 °C for 10 s, 56 °C for 10 s, and 72 °C for 30 s, with a final extension at 72 °C for 5 min [57]. The purified amplicons were sequenced by MAGIGENE Biological Technology Co. Ltd., Guangzhou, China, and taxonomic identities were determined using BLAST at www.ncbi.nlm.nih.gov/blast/Blast.cgi (accessed on 10 October 2023). The bacterial isolates exhibiting > 97% 16S rRNA gene similarity to ASVs remarkably enriched in both bulk soil and rhizosphere were selected, obtaining five enriched bacterial strains for further study.

### 4.4. Glasshouse Pot Experiments

To assess the impact of the SynCom on the growth and P uptake of rice, natural bulk soil was collected from the agricultural field. The treated soils were sterilized with X-ray radiation exposure (50 kGy) at Shanghai Co-Elit Agricultural Sci Tech Co., Shanghai, China, and then filled into pots (14 cm deep and 10 cm in diameter) with 200 g of soil per pot. Rice seeds were germinated at 28 °C for 10 days after being surface sterilized with 1% (*v*/*w*) sodium hypochlorate for 5 min. Each pot received one transplanted 10-day-old rice seedling. Five days after transplanting, bacteria were cultured in their respective liquid media on a shaker (180 rpm, 28 °C) for two days and harvested by centrifugation (5000× *g*, 10 min). The concentration of the cultured bacterial strain was adjusted to 1 × 10^8^ CFU/g with sterilized water. A 15 mL suspension of this SynCom was injected into the sterilized soil; sterilized water served as a control. All pots were randomly placed in a greenhouse (32 °C day/22 °C night, relative humidity of 60–80%, 16 h light/8 h dark). The rice samples were taken at 25 days post-inoculation.

### 4.5. Soil Nutrient and Root System Architecture Analysis

The effect of SynCom inoculation on soil nutrient levels was assessed. The collected soil samples were air-dried, ground, and sieved through a 2 mm mesh to remove oversized particles [58]. Subsequently, the physicochemical characteristics were extracted in accordance with China National Standard Methods (www.chinesestandard.net). Briefly, soil pH was determined in a suspension with 1:2.5 soil/water (*w*/*v*) using a pH meter (LY/T1239-1999) [59]. Electrical conductivity (EC) was measured in a suspension with 1:2.5 soil/water (*w*/*v*). Soil total N contents (TN) and total N contents (TC) contents were measured by elemental analyzer (vario Macro Cube, Elementar, Hanau, Germany). Available potassium (AK) was extracted with 1 mol L^−1^ ammonium acetate solution buffered at pH 7.0 and then measured using a flame photometer (NY/T 889-2004) [60]. NO_3_^−^-N and NH_4_^+^-N contents were determined by AA3 automated flow injection analysis (SEAL Analytical GmbH, Norderstedt, Germany). The P fractions in the soil were determined by the Hedley sequential fractionation method, as modified by Tiessen [61,62] The sequential extraction procedure involved sequentially extracting 0.5 g of soil with 30 mL of water, 0.5 M NaHCO_3_ (pH 8.5), 0.1 M NaOH, and 1 M HCl shaking at 200 rpm and 25 °C for 16 h. Stratified soil P measurements were performed by sequential extraction to quantify the amount of available P, primary P, secondary P, organic P, and residual P.

For root distribution analysis, rice seedlings were transplanted and grown in column systems in a greenhouse using 2.5 kg of sterilized soil per column (40 cm in height × 10 cm in diameter) under dry farming conditions, with one plant per column. The planting and SynCom inoculation conditions followed those used in the pot trials. Rice samples were collected 25 days post-inoculation, and the soil was separated into four layers (0–10 cm, 10–20 cm, 20–30 cm, and 30–40 cm). All visible roots in each layer were carefully collected by hand, washed, and prepared for further analysis. For root morphology analysis, rice plants were grown in 1 L pots, and samples were taken 25 days post-inoculation. The entire root system was collected and washed for subsequent analyses. Cleaned roots were placed in a Petri dish filled with a 5 to 6 mm layer of water and spread out with tweezers to minimize overlap, and then scanned at 600 DPI using a flatbed scanner (Perferction V700 Photo; Epson America). Total root length, root surface area, average diameter and tip number were then analyzed using the image-processing software WinRhizo Pro v2019a (Regent Instruments, Québec, Canada).

### 4.6. Gene Expression Analysis

To assess the impact of SynCom inoculation on genes related to root-to-shoot Pi translocation in rice roots, we quantified expression of PHT1 family transporters. Total RNA extraction was conducted using the RNAprep Pure Plant Kit (TIANGEN, Beijing, China). Subsequently, 300 nanograms of each total RNA sample were reverse transcribed using the PrimerScript RT Master Mix (Takara, Dalian, China). Quantitative reverse transcription PCR (QRT-PCR) assays were conducted on a LightCycler^®^ 96 Instrument (Roche, Switzerland) with an SYBR Green I Master (Roche Diagnostics GmbH, Mannheim, Germany). The housekeeping gene *OsACTIN* served as an internal standard for normalizing cDNA concentrations. Relative gene expression levels were calculated using the 2^−ΔΔCt^ method. The primers employed for QRT-PCR of all tested genes are listed in Table S2.

### 4.7. Statistical Analysis

Differences in plant biomass, P uptake, P fractions, gene expression and root morphology were analyzed using analysis of variance (ANOVA) followed by pairwise comparisons of Least Square Means (LSMeans). The False Discovery Rate (FDR) method was applied for correction. Subsequent to ANOVA, post hoc tests were conducted with FDR correction. Model validation involved scrutinizing residuals and testing for overdispersion. The entire statistical analyses were performed using R v4.2.0 and involved packages including “lme4 v1.1.13”, “car v3.1.2”, “emmeans v1.10.1”, “multcomp v1.4.25” and “RVAideMemoire v0.9.83.7”.

## 5. Conclusions

Our findings indicated that interspecific competition in the rice/soybean intercropping system significantly altered the soil microbiome composition of the complementer, which is rice. By applying a SynCom enriched with bacteria from intercropping system, enhanced P uptake in rice could be accomplished by increasing available P in soil, optimizing root architecture, and promoting root-to-shoot P translocation. We provide evidence that rice plants coordinate with the root microbiome to acquire P under interspecific competition, supporting optimal plant growth. Harnessing microbial strategies, such as a specialized SynCom, is a promising microbial tool for agricultural sustainability to improve plant yield and nutrient acquisition.

## Data Availability

The sequences obtained in this study have been deposited in the in the Genome Sequence Archive (Genomics, Proteomics & Bioinformatics 2021) in National Genomics Data Center, China National Center for Bioinformation / Beijing Institute of Genomics, Chinese Academy of Sciences (GSA: CRA020594) that are publicly accessible at https://ngdc.cncb.ac.cn/gsa.

## Supplementary Materials

The following supporting information can be downloaded at: https://www.mdpi.com/article/10.3390/ijms252312819/s1.
