# Supplementary material for: Synthetic Microbial Community Isolated from Intercropping System Enhances P Uptake in Rice
Source: Int J Mol Sci. 2024 Nov 28;25(23):12819. doi: 10.3390/ijms252312819 (PMC11641191; doi:10.3390/ijms252312819)
Supplement: Supplementary file 1 [file ijms-25-12819-s001.zip › ijms-3336212-supplementary.pdf]

Supplementary Materials for

Specific bacterial consortium isolated from intercropping system enhances P uptake in rice

Huimin Ma, Hongcheng Zhang, Congcong Zheng, Jing Wang, Zhihai Wu, Hualiang Zhang

Correspondence author: wuzhihai@jlau.edu.cn; hualiangzhang@zju.edu.cn

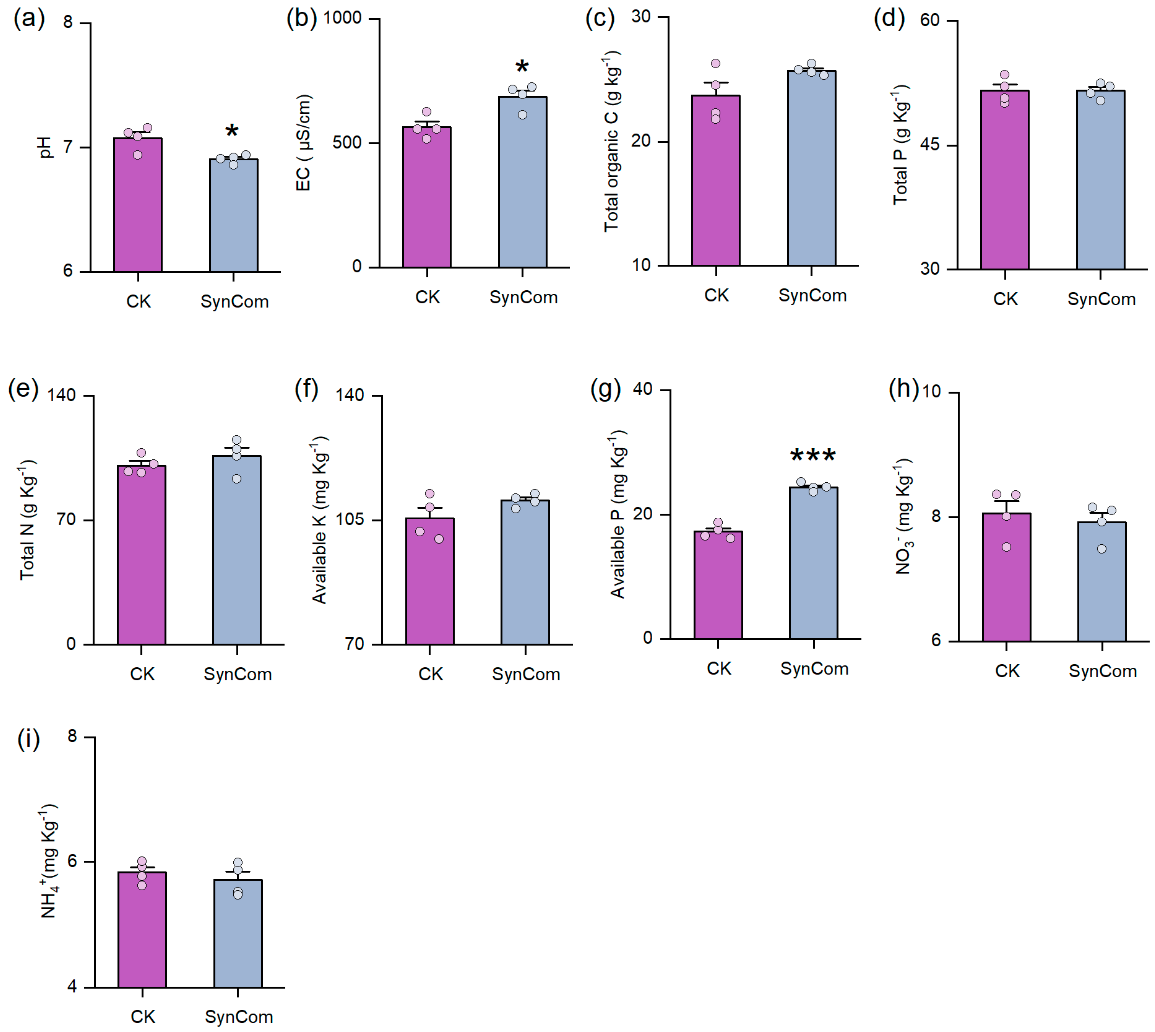

Figure S1. Nutrient contents in rhizosphere soils inoculated with SynCom and with sterilized water. The soil pH (**a**), EC (**b**), total organic carbon (**c**), total P (**d**), total N (**e**), available K (**f**), available P (**g**), NO_3^-(**h**) and NH_4^+(**i**) in rhizosphere soil of rice plants after inoculated bacterial synthetic community (SynCom) and sterilized water (CK). n = 4 biological replicates were measured for all the treatment. Data are average ± s.e.m. Data points represent individual replicate samples. Asterisks indicate significant differences between treatments (ANOVA, FDR-corrected LSMeans, *$P < 0.05$; **$P < 0.01$; ***$P < 0.001$).

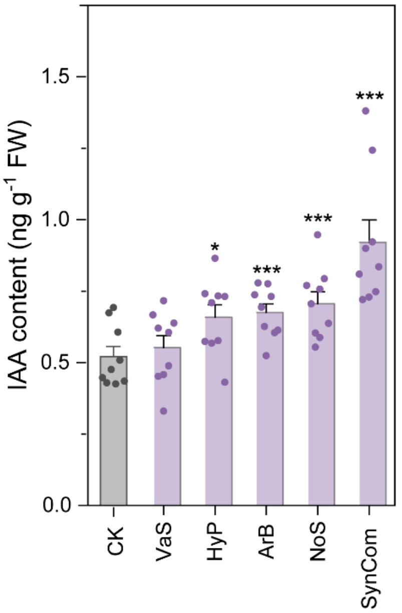

Figure S2. Special enriched bacterial strains and SynCom affect IAA content in rice root. CK, Sterilized water; VaS, *Variovorax_paradoxus*; HyP, *Hydrogenophaga_pseudoflava*; ArB, *Acidovorax_sp*.; NoS, *Novosphingobium_subterraneum*; SynCom, bacterial synthetic community (SynCom). Data are average ± s.e.m. Data points represent individual replicate samples. n = 9 biological replicates were measured for all the treatment. Asterisks indicate significant differences between treatments (ANOVA, FDR-corrected LSMeans, **P* < 0.05; ****P* < 0.001).

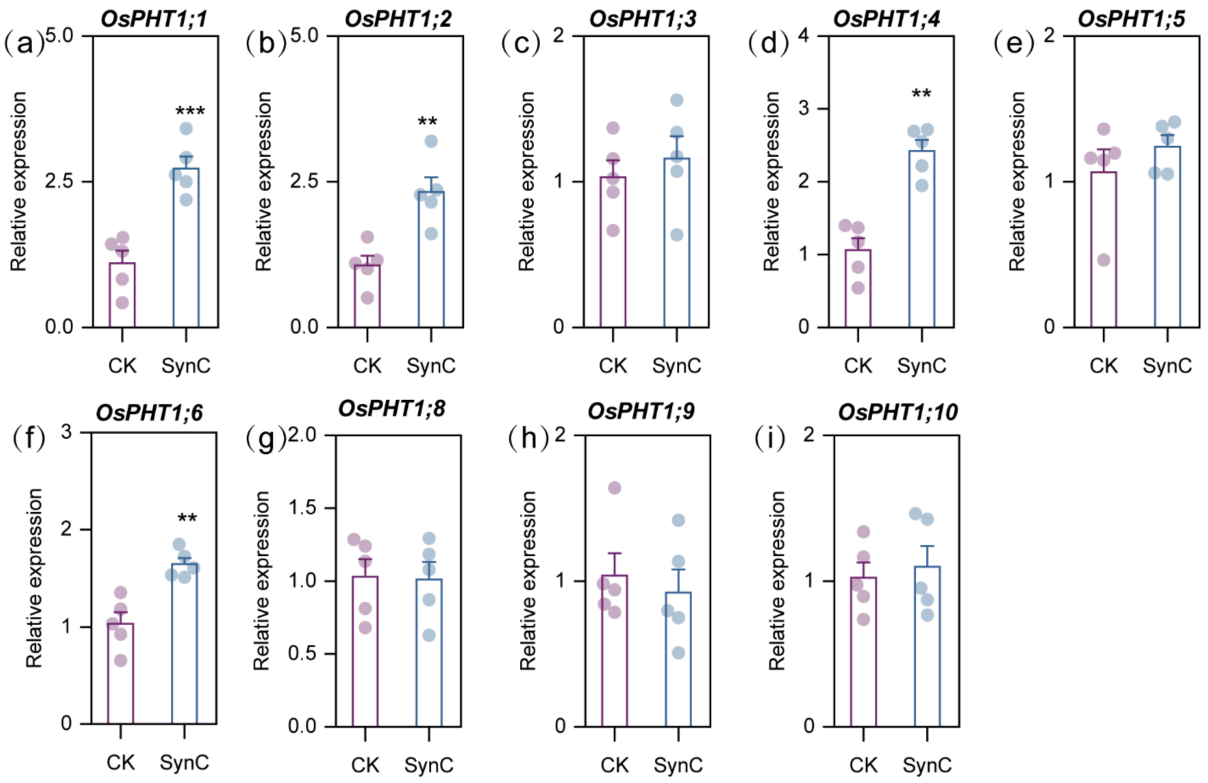

Figure S3. SynCom regulates the expression of Pi transporter genes. The expression levels of *OsPHT1;1* (**a**), *OsPHT1;2* (**b**), *OsPHT1;3* (**c**), *OsPHT1;4* (**d**), *OsPHT1;5* (**e**), *OsPHT1;6* (**f**), *OsPHT1;7* (**g**), *OsPHT1;8* (**h**), *OsPHT1;9* (**i**), *OsPHT1;10* (**j**) in rice plants inoculated sterilized water (CK) or bacterial synthetic community (SynC). n = 10 biological replicates were measured for all the treatment. Data are average ± s.e.m. Data points represent individual replicate samples. Asterisks indicate significant differences between treatments (ANOVA, FDR-corrected LSMeans, **$P < 0.01$; ***$P < 0.001$).

Supplemental Table S1. Topological features of co-occurrence networks. Bacterial co-occurrence networks of rice plants in bulk and rhizosphere after rice monoculture and rice/soybean intercropping.

**Properties**	**Bacteria**			
	**Bulk**		**Rhizosphere**	
	**RM**	**IRS**	**RM**	**IRS**
**Average degree**	19	18.317	15.426	14.074
**Average path length**	2.306	2.32	2.662	2.582
**Average Weighted Degree**	7.417	**9.495**	9.32	4.732
**Density Plot**	0.184	0.183	0.154	0.134
**Clustering coefficient**	0.564	0.566	0.605	0.536
**Network diameter**	7	5	6	6
**Modularity**	0.669	0.464	0.38	**0.833**
**Number of edges**	988	925	779	760
**Nodes**	104	101	101	**108**

Supplemental Table S2 Gene ID and primer sequences of genes for RT-qPCR.

Gene name	Gene ID	Forward primer (5'-…-3')	Reverse primer (5'-…-3')
OsPHT1;1	Os03g0150600	CGCTTCCGTACGAGTGGTA GT	GGTTCTTTCAAATCCAGGGAAA
OsPHT1;2	Os03g0150800	GACGAGACCGCCCAAGAA G	TTTTCAGTCACTCACGTCGAGAC
OsPHT1;3	Os10g0444600	TGCGACTGCTGTATTCAGT ACGT	ACAAATGCCATCAAATATGAACAGA
OsPHT1;4	Os04g0186400	TATTGCGGCTTAGATTGCA TTAG	TCCAAATCAAATGGGCACTAAG
OsPHT1;5	Os04g0185600	TGCTACTGCCCATGACTAG GATT	CCATAGAAGAGATCCAGAGAAGCT GTA
OsPHT1;6	Os08g0564000	CCGCCCCTGCAAACTGTA	GAACTGGCGGTTTCTTCGAT
OsPHT1;7	Os03g0136400	GCAAGTCGCTCGAGGAGA TG	TGGAATTAACGGGTGGATCAC
OsPHT1;8	Os10g0444700	AGAAGGCAAAAGAAATGT GTGTTAAAT	AAAATGTATTCGTGCCAAATTGCT
OsPHT1;9	Os06g0324800	AGAAAAACATAGGCTTGT CATCCTTT	AAAACCTAAGAAGCACTGTAAATA AATCC
OsPHT1;10	Os06g0325200	ATGTCGCCCATCCTTCCA	TCGCTTTCCGACGATGATC
OsPHT1;11	Os01g0657100	GAGAAGTTCCCTGCTTCAA GCA	TGCATATCCCAGATGAGCGTATC
OsPHT1;12	Os03g0150500	AACGAGATGACGAACACT TGCA	TCCTGTACCTAAAAAGCAAGTACTA ACATAGT
OsPHT1;13	Os04g0186800	TTAGCATAATTCTTTTAGT AGTTAAATAGGAGATG	TGATTTAAGATAAGGATTGAATGCA CAT
OsACTIN	Os03g50885	TGGACAGGTTATCACCATT GGT	CCGCAGCTTCCATTCCTATG
